# The Salford Lung Study protocol: a pragmatic, randomised phase III real-world effectiveness trial in asthma

**DOI:** 10.1186/s12890-015-0150-8

**Published:** 2015-12-10

**Authors:** Ashley Woodcock, Nawar Diar Bakerly, John P. New, J. Martin Gibson, Wei Wu, Jørgen Vestbo, David Leather

**Affiliations:** Institute of Inflammation and Repair, Manchester Academic Health Science Centre, University of Manchester, Manchester, UK; Salford Royal NHS Foundation Trust, Salford, UK; GlaxoSmithKline, Research Triangle Park, Durham, NC USA; GSK Respiratory Centre of Excellence, GlaxoSmithKline UK Ltd, Uxbridge, UK

**Keywords:** Asthma, Electronic medical record, Fluticasone furoate/vilanterol, Inhaled corticosteroid/long-acting β_2_-agonist, Pragmatic randomised controlled trial, Real-world evidence

## Abstract

**Background:**

Novel therapies need to be evaluated in normal clinical practice to allow a true representation of the treatment effectiveness in real-world settings.

**Methods/design:**

The Salford Lung Study is a pragmatic randomised controlled trial in adult asthma, evaluating the clinical effectiveness and safety of once-daily fluticasone furoate (100 μg or 200 μg)/vilanterol 25 μg in a novel dry-powder inhaler, versus existing asthma maintenance therapy. The study was initiated before this investigational treatment was licensed and conducted in real-world clinical practice to consider adherence, co-morbidities, polypharmacy, and real-world factors. Primary endpoint: Asthma Control Test at week 24; safety endpoints include the incidence of serious pneumonias. The study utilises the Salford electronic medical record, which allows near to real-time collection and monitoring of safety data.

**Discussion:**

The Salford Lung Study is the world’s first pragmatic randomised controlled trial of a pre-licensed medication in asthma. Use of patients’ linked electronic health records to collect clinical endpoints offers minimal disruption to patients and investigators, and also ensures patient safety. This highly innovative study will complement standard double-blind randomised controlled trials in order to improve our understanding of the risk/benefit profile of fluticasone furoate/vilanterol in patients with asthma in real-world settings.

**Trial registration:**

Clinicaltrials.gov, NCT01706198; 04 October 2012.

## Background

Combination of a long-acting inhaled corticosteroid (ICS) fluticasone furoate (FF) with the novel long-acting β_2_-agonist (LABA) vilanterol (VI) in a novel dry-powder inhaler (DPI; Ellipta^®^) has been investigated as a once-daily medication for the management of asthma [1]. Following a phase III programme of randomised controlled trials (RCTs), marketing authorisation was given by the European Commission on 18 November 2013 and FF/VI (Relvar^®^) is now licensed across Europe for asthma and chronic obstructive pulmonary disease.

We have previously described the principles behind the Salford Lung Study (SLS) and how this pragmatic RCT (pRCT) differs from standard RCTs [2]. The study was originally designed and approved before the investigational treatment received regulatory approval, and hence is a phase III study. It compares the FF/VI combination with existing maintenance therapy, in a large population of patients with asthma, studied in real-world routine clinical practice, and monitored using an electronic medical record (EMR). Here we provide details of the SLS asthma protocol.

## Methods

### Study design

**Fig. 1:**
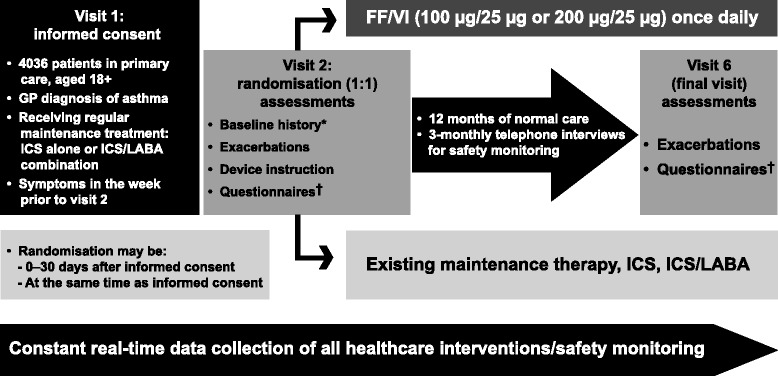
Study design. *Cardiovascular risk factors collected. ^†^Comprises: Asthma Control Test; Asthma Quality of Life Questionnaire(s); EuroQol questionnaire; Medication Adherence Report Scale for Asthma; Work Productivity and Activity Impairment Questionnaire: Asthma. *FF* fluticasone furoate; *GP* general practitioner; *ICS* inhaled corticosteroid; *LABA* long-acting β_2_-agonist; *VI* vilanterol

SLS is a 12-month randomised, open-label, phase III pRCT evaluating the effectiveness and safety of FF/VI (Relvar^®^; 100 μg/25 μg or 200 μg/25 μg once daily, delivered by a novel DPI [Ellipta^®^] in patients with asthma) (clinicaltrials.gov identifier NCT01706198) (Fig. 1). The study is conducted in accordance with the International Conference on Harmonisation, Good Clinical Practice (GCP), all applicable data protection requirements and the ethical principles outlined in the Declaration of Helsinki 2013 (National Research Ethics Service Committee North West, Greater Manchester South; Research Ethics Committee reference 12/NW/0455). The study protocol conforms to the SPIRIT 2013 statement (Standard Protocol Items: Recommendations for Interventional Trials [3, 4]).

The Salford Lung Study team sought guidance on the study design under the joint scientific advice process from National Institute for Health and Care Excellence and the Medicines and Healthcare Products Regulatory Agency; a joint consultation process took place to seek guidance on the study design. Informal discussions and advice on the study design took place prior to formal ethics application from the National Research Ethics Service Committee North West, Greater Manchester South.

### Patients

All patients with asthma at 66 primary care sites (at the time of manuscript preparation) in and around Salford and South Manchester are identified from practice databases, and invited to participate in the study by their own general practitioner (GP) (Fig. 1).

Eligibility criteria include:

- aged ≥18 years
- symptomatic asthma diagnosed by a GP
- regular maintenance inhaler therapy with ICS or ICS/LABA
- symptoms within the week prior to visit 2.

There are minimal exclusion criteria such as a recent history of life-threatening asthma, chronic obstructive pulmonary disease or other clinically significant disease that would jeopardise patient safety. Eligible patients are recruited for the study in GPs’ offices. At visit 1, patients are offered study participation through written consent. At visit 2 (0–30 days after visit 1), patients are randomised 1:1 to receive FF/VI or continue on usual asthma maintenance therapy (ICS or ICS/LABA). The randomisation at visit 2 is stratified by Asthma Control Test (ACT) score (≥20, 16 to 19, or ≤15) and by previous asthma maintenance therapy (ICS or ICS/LABA). Patients randomised to continue their existing asthma maintenance therapy arm do not receive FF/VI. During the study, the doses of all medication may be adjusted at the GP’s discretion in the usual way. Both study groups receive free prescriptions for study medication, which is collected by the patients from local community pharmacies, and prescription data are captured on the electronic case report forms (eCRFs).

### Participating sites

#### Primary care

To preserve the real-world nature of the study, the patient experience is as close to routine care as possible. The study’s principal investigators are the patients’ own GPs who may make treatment adjustments according to their clinical opinion. GPs make repeat prescriptions of study medication as usual, which are collected by patients from their usual pharmacy. GPs are ideally placed to facilitate recruitment, identify and report serious adverse events (SAEs) or serious adverse drug reactions (ADRs), and report study endpoints. As very few participating GPs had experience of clinical trial participation, all GPs have received training and support in GCP, patient recruitment, the study protocol, coding of healthcare issues and general research procedures.

#### Pharmacy

Every pharmacy in Salford and others in South Manchester have agreed to participate in the study, even though very few of the pharmacists had experience of clinical trial participation. Standard operating procedures were established, and more than 500 staff at participating pharmacies have received training in GCP and safety reporting. Initially pharmacies faxed copies of all prescriptions for collected study treatments to the study coordination centre, but as the trial progressed this has been collected electronically.

#### Hospitals

The large majority of admissions are to the local Salford Royal Hospital and the University Hospital of South Manchester where admissions are tracked electronically and in near-real time. Occasionally patients are admitted to other hospitals. These admissions are tracked via the primary care records. Information relating to all hospitalisations is reviewed by a dedicated study safety team.

### Data monitoring

All hospital admissions, outpatient and emergency department visits are identified from the EMR database (whenever and wherever they occur). From primary care, all healthcare contacts, out-of-hours activity and prescriptions of antibiotics or oral steroids can be identified. These events are reviewed by the study research team and classified as asthma or non-asthma related. Furthermore, the EMR captures suspected unexpected serious adverse reactions (e.g. reduced kidney function or elevated liver function tests) and, for the purposes of SLS, includes data from external sources to identify, for example, deaths or National Health Service (NHS) hospital admissions outside Salford. Northwest EHealth [5] manages the EMRs, enabling data on study endpoints and patient safety to be collected continuously and remotely in near-real time.

### Endpoints

#### Efficacy

The primary endpoint is the percentage of patients in each treatment arm, who have *either* an ACT total score of ≥20 *or* an increase from baseline of ≥3 in ACT total score at week 24 assessment. Secondary efficacy endpoints include: the percentage of patients who have either an ACT total score of ≥20 or an increase from baseline of ≥3 in ACT total score at weeks 12, 40 and 52; the mean change from baseline in ACT total score at weeks 12, 24, 40 and 52; and the percentage of patients with an ACT total score ≥20 at weeks 12, 24, 40 and 52. Further details of trial endpoints can be found in Table 1. Randomisation is stratified by baseline asthma therapy (ICS or ICS/LABA) and ACT score (≤15, 16–19, ≥20) to ensure treatment groups are balanced on disease severity and level of asthma control. These randomisation stratification variables will be included in the primary analysis model as covariates. Subgroup summaries and/or analysis will also be provided by the randomisation stratification variables, when appropriate.

**Table 1:** Study endpoints

Endpoint	Definitions
Primary endpoint	The primary efficacy analysis population is defined as all ITT patients who have an ACT total score of <20 at baseline
The percentage of patients who have either an ACT total score of ≥20 or an increase from baseline of ≥3 at week 24 (6th month) assessment	
Secondary efficacy endpoints	• All contacts are any encounter the patient may have with a doctor or nurse or other healthcare professionals working as part of the NHS (including telephone calls). Contacts with the NHS or hospitalisation are defined as exacerbation-related contacts if these contacts were a direct result of an acute worsening of asthma symptoms. Contacts are defined to be asthma-related as per GP/investigator-defined normal clinical practice. These contacts do not include protocol-defined study-related visits/contacts • A prescription of systemic corticosteroid or antibiotics is defined as exacerbation-related if the reason the drug was given, in whole or in part, was to treat an acute worsening of asthma symptoms • A severe asthma exacerbation is defined as deterioration of asthma requiring the use of systemic corticosteroids (tablets, suspension or injection) or antibiotics, an inpatient hospitalisation, or emergency department visit due to asthma that required systemic corticosteroids or antibiotics. Exacerbation-related hospitalisation includes hospitalisation that is prolonged as a result of an asthma exacerbation
• Percentage of patients who have either an ACT total score of ≥20 or an increase from baseline of ≥3 in ACT total score at weeks 12, 40 and 52 • Percentage of patients with ACT total score ≥20 at weeks 12, 24, 40 and 52 • Mean change from baseline in ACT total score at weeks 12, 24, 40 and 52 • All/asthma-related primary or secondary care contacts • Mean annual rate of severe asthma exacerbations • Number of salbutamol inhalers (adjusted to equivalence of 200 actuations) dispensed from study-enrolled community pharmacies over the entire treatment period • Time to discontinuation or modification of initial therapy (i.e. therapy the patient is randomised to at visit 2) • Percentage of patients who have an increase from baseline of ≥0.5 in AQLQ(s) total score at week 52 • Percentage of patients who have an increase from baseline of ≥0.5 in AQLQ(s) environmental stimuli domain score at week 52	
Safety endpoints	• An ADR is any untoward medical occurrence in a patient temporally associated with the use of a medicinal product, for which there is a reasonable possibility that the untoward occurrence is causally related to the medicinal product • An SAE is any untoward medical occurrence that, at any dose: results in death; is life-threatening; requires hospitalisation or prolongation of existing hospitalisation; results in disability/incapacity; is a congenital anomaly/birth defect; subject to medical or scientific judgement, may jeopardise the patient or may require medical or surgical intervention to prevent one of the other outcomes listed
• Incidence of SAEs of pneumonia during the study • Time to first SAE of pneumonia • Deaths due to serious events of pneumonia • Frequency and type of SAEs • Frequency and type of ADRs	
Other efficacy endpoints	• Increase from baseline of ≥0.5 in AQLQ(s) refers to: total score at week 24; individual domain scores at week 24; individual domain scores (symptoms, activity, limitations and emotional function) at week 52 • WPAI: asthma at week 52 refers to the following categories: percentage of work time missed due to asthma: percentage of impairment while working due to asthma; percentage of overall work impairment due to asthma; percentage of activity impairment due to asthma
• Mean change from baseline in individual question scores for ACT at weeks 12, 24, 40 and 52 • Mean change from baseline in total score and domain scores of AQLQ(s) at weeks 24 and 52 • Percentage of patients who have an increase from baseline of ≥0.5 in AQLQ(s) • WPAI: asthma at week 52 • Health status using the EQ-5D at week 52 • Adherence with study medication based on analysis of medications (prescribed, dispensed and collected) during the study • Use of the MARS-A at week 52	

*ACT* Asthma Control Test, *ADR* adverse drug reaction, *AQLQ* Asthma Quality of Life Questionnaire, *EQ*-5D EuroQol questionnaire, *GP* general practitioner, *ITT* intent-to-treat, *MARS*-*A* Medication Adherence Report Scale for Asthma, *NHS* National Health Service, *SAE* serious adverse event, *WPAI* Work Productivity and Activity Impairment Questionnaire

#### Safety

Safety endpoints include the frequency and type of SAEs and ADRs, and the incidence of SAEs of pneumonia during the study. SAEs are monitored continually through the patient’s EMR. GPs/investigators or site staff are responsible for detecting, documenting and reporting SAEs and non-serious ADRs, identified by hospitalisation alerts through EMR and reported on eCRFs, with additional monitoring by telephone every 3 months.

### Statistical analysis

A sample size of 2906 patients (1453 patients per treatment group) will detect a treatment difference of 6 % between usual asthma maintenance therapies and FF/VI on the primary endpoint, at the significance level 0.05 and 90 % power (assuming 50 % response rate in the usual asthma maintenance group at 6 months). A total of 4036 patients are required in the intent-to-treat (ITT) population (randomisation of 2018 patients per treatment arm) in order to have at least 2906 patients in the primary efficacy analysis population, assuming 80 % of patients in the ITT population have an ACT score of <20 at baseline and a 10 % dropout rate over the first 6-month period.

Primary efficacy analysis population is all ITT patients, who have an ACT total score <20 at baseline (randomisation visit). Treatment difference between the two treatment arms will be analysed using logistic regression adjusting for baseline ACT total score, baseline asthma therapy per randomisation stratification (ICS or ICS/LABA), age and gender. Subgroup analyses, when appropriate, will be provided for efficacy and safety endpoints based on baseline disease characteristics per randomisation stratification.

## Discussion

Guidelines on treatment options are primarily based on double-blind RCTs (DBRCTs) [6, 7]. However, RCTs for registration purposes do not represent real life. Multiple inclusion and exclusion criteria mean that only a small proportion of patients with asthma (~5 %) are represented in DBRCTs, and patients with co-morbidities are excluded. In addition, only restricted outcomes are assessed (e.g. forced expiratory volume in 1 second), and studies are of short duration (<6 months). Finally, patients are closely monitored and inhaler technique repeatedly checked, so that adherence levels exceed 90 %, compared with less than 40 % in observational studies.

Although observational studies provide opportunities to assess real-world outcomes, the lack of suitable comparison between treatment groups and the small patient numbers in prospective trials can be limiting [8]. Consequently, the true impact and value of treatments for asthma may not be fully reflected by observational studies and DBRCTs.

The SLS is the first phase III pRCT study in asthma initiated while the investigational treatment was an un-licensed medicine, where supervision and monitoring of patients are reduced to a minimum. Clinical endpoints are collected and patient safety is guaranteed with remote clinical surveillance in near to real-time through the EMR. The study should provide evidence for prescribers, payers and healthcare providers to assess effectiveness, adherence levels and the true value of FF/VI treatment in the real world.

The primary endpoint, ACT, was chosen to reflect impact of treatments on patients’ overall asthma control. The rate of severe asthma exacerbations as a primary endpoint would not have been feasible due to the infrequent occurrence of such events in a general asthma population [9]; consequently, the number of eligible patients with asthma in the study population is insufficient to have adequate statistical power for an exacerbation study. However, although ACT may reflect a dimension of asthma that is different from exacerbations, it is not merely a proxy for exacerbations.

This pRCT has been a major challenge in study design, operational planning and study support. It has been possible because the SLS is focused on a single geographical area in the UK, with a stable population with high respiratory morbidity, and where healthcare is managed by the NHS. Salford and the surrounding area are unique in having a longstanding EMR connecting primary and secondary care together with local pharmacies. The extent to which these data will be transferable will depend on local health service provision, but SLS should provide a model for future assessments of the clinical effectiveness of new treatments.

In summary, data from the SLS will complement results from conventional registration trials, allowing a better understanding of the risk/benefit profile of the FF/VI combination in the wider community of patients with asthma.

## Abbreviations

ACT: Asthma Control Test
ADR: Adverse drug reaction
AQLQ: Asthma Quality of Life Questionnaire
DBRCT: Double-blind randomised controlled trial
DPI: Dry-powder inhaler
eCRF: Electronic case report form
EMR: Electronic medical record
EQ-5D: EuroQol questionnaire
FF: Fluticasone furoate
GCP: Good Clinical Practice
GP: General practitioner
ICS: Long-acting inhaled corticosteroid
ITT: Intent-to-treat
LABA: Long-acting β_2_-agonist
MARS-A: Medication Adherence Report Scale for Asthma
NHS: National Health Service
pRCT: pragmatic randomised controlled trial
RCT: Randomised controlled trial
SAE: Serious adverse event
SLS: Salford Lung Study
VI: Vilanterol
WPAI: Work Productivity and Activity Impairment Questionnaire
